# How much do time-domain functional near-infrared spectroscopy (fNIRS) moments improve estimation of brain activity over traditional fNIRS?

**DOI:** 10.1117/1.NPh.10.1.013504

**Published:** 2022-10-22

**Authors:** Antonio Ortega-Martinez, De’Ja Rogers, Jessica Anderson, Parya Farzam, Yuanyuan Gao, Bernhard Zimmermann, Meryem A. Yücel, David A. Boas

**Affiliations:** Boston University Neurophotonics Center, Boston, Massachusetts, United States

**Keywords:** functional near-infrared spectroscopy, time-domain, moments, root-mean-square error, correlation, general linear model

## Abstract

**Significance:**

Advances in electronics have allowed the recent development of compact, high channel count time domain functional near-infrared spectroscopy (TD-fNIRS) systems. Temporal moment analysis has been proposed for increased brain sensitivity due to the depth selectivity of higher order temporal moments. We propose a general linear model (GLM) incorporating TD moment data and auxiliary physiological measurements, such as short separation channels, to improve the recovery of the HRF.

**Aims:**

We compare the performance of previously reported multi-distance TD moment techniques to commonly used techniques for continuous wave (CW) fNIRS hemodynamic response function (HRF) recovery, namely block averaging and CW GLM. Additionally, we compare the multi-distance TD moment technique to TD moment GLM.

**Approach:**

We augmented resting TD-fNIRS moment data (six subjects) with known synthetic HRFs. We then employed block averaging and GLM techniques with “short-separation regression” designed both for CW and TD to recover the HRFs. We calculated the root mean square error (RMSE) and the correlation of the recovered HRF to the ground truth. We compared the performance of equivalent CW and TD techniques with paired t-tests.

**Results:**

We found that, on average, TD moment HRF recovery improves correlations by 98% and 48% for HbO and HbR respectively, over CW GLM. The improvement on the correlation for TD GLM over TD moment is 12% (HbO) and 27% (HbR). RMSE decreases 56% and 52% (HbO and HbR) for TD moment compared to CW GLM. We found no statistically significant improvement in the RMSE for TD GLM compared to TD moment.

**Conclusions:**

Properly covariance-scaled TD moment techniques outperform their CW equivalents in both RMSE and correlation in the recovery of the synthetic HRFs. Furthermore, our proposed TD GLM based on moments outperforms regular TD moment analysis, while allowing the incorporation of auxiliary measurements of the confounding physiological signals from the scalp.

## 1 Introduction

Functional near-infrared spectroscopy (fNIRS) is a technology that measures functional changes in the hemodynamics of the brain.^1^ In its simplest form, near-infrared sources and detectors are placed on the scalp, and changes in the optical properties of the tissue produced by hemodynamic activity are detected as changes in the detected light intensity. The technology is widely used in cognitive studies,^2^^,^^3^ for populations that cannot use fMRI, as well as for other applications, such as brain–computer interfaces^4^ or clinical diagnosis.^5^ The most common mode of fNIRS, in which light is emitted and detected continuously, is known as continuous wave fNIRS (CW fNIRS). Although this type of setup is very easy to implement, it has the disadvantage that it is not always easy to distinguish when changes in intensity are produced by hemodynamics of the brain or by other physiology, e.g., by hemodynamic changes in the scalp or motion artifacts.^6^^,^^7^ One of the reasons for this is that all photons reaching the detector are modulated by skin hemodynamics produced by respiration and the cardiac pulse. Furthermore, CW fNIRS detectors integrate photons from a large number of paths in the tissue, many of which did not reach the brain, reducing the contrast to brain ratio.

Several solutions have been proposed and implemented over the years to improve the contrast of the brain signal in fNIRS. The simplest approach is to subject the user to multiple repetitions of a task or a stimulus at random intervals and then perform an average of the time intervals around the stimulus. Another more sophisticated method is the general linear model (GLM), which involves least-squares fitting of the measured signal to a linear model incorporating additional knowledge of the signal in the form of auxiliary signals [e.g., short separation (SS) channels to model the systemic physiology^8^]. However, the low sensitivity to the brain layers of CW fNIRS, along with statistical properties of the signal violating assumptions of the GLM^9^ can still complicate the interpretation of the neural signals.

Time-domain fNIRS (TD fNIRS) is an alternative to CW fNIRS which offers improved sensitivity to the brain hemodynamics.^10^^,^^11^ Although TD fNIRS was traditionally associated with bulky and expensive equipment, advances in electronics have allowed the recent development of compact, high-channel count systems.^12^ TD fNIRS uses time gating to discriminate the photons arriving to the detector as a function of their time of flight. As photons traveling longer distances are more likely to have reached deeper layers of the tissue, TD-fNIRS has increased sensitivity to the brain hemodynamics when longer-traveling photons are selected. However, this advantage over CW fNIRS is limited in real instrumentation by the instrument response function (IRF), which causes a broadening of the distribution of times of flight (DTOF), complicating the interpretation of the time gates. Moment analysis of the DTOF,^13^ which is relatively immune to the IRF,^14^ has been proposed as an alternative to time gates analysis in TD-fNIRS. Higher statistical moments of the DTOF present increased sensitivity to deeper tissue layers compared to signal intensity changes as the kernel for the moment calculation grows as a function of the time of flight.^15^ Nevertheless, TD moment analysis reported in the literature still relies on block averaging to recover the hemodynamic response function (HRF) which, unlike the GLM, does not allow for the incorporation of additional knowledge of the shape of the HRF or the confounds in the signal.

In this work, we compare the performance of moment TD fNIRS to equivalent techniques using only CW data in recovering the HRF from resting time series data augmented with a known synthetic HRF. We also propose a GLM formulation to perform linear regression with moment data to permit us to better distinguish the HRF from confounding physiological interference in the scalp by incorporating auxiliary measurements, such as SS channels. We evaluate the performance of the methods by comparing the resultant root-mean-square error (RMSE) and the correlation of the recovered HRF. We show that proper covariance weighting of the TD moments data is needed to obtain optimal estimation of the HRF. We also find that the optimal variance weighting is not the traditionally described shot-noise-dependent covariance of the moments.

## 2 Theory

The n’th moment of the DTOF for n>0 is given by^16^

$$m_{n}=\frac{1}{m_{0}}\underset{i}{\sum }\tau_{i}^{n}N_{i}.$$ (1)

$N_{i}$ is the height of the i’th bin of the DTOF (i.e., the number of photons in that bin), $\tau_{i}$ is the time of flight associated with the i’th bin, and $m_{0}$ is the zeroth moment of the DTOF given by $\sum_{i}N_{i}$. For this work, we will define $M_{0}=-\log(m_{0})$, the logarithm of the area under the curve of the DTOF for a given time sample (equivalent to the optical density used in CW fNIRS analyses), $M_{1}=m_{1}$, the mean time of flight; and $M_{2}=m_{2}-m_{1}^{2}$, the variance of the DTOF. During this work, we will refer to $M_{0}$, $M_{1}$, and $M_{2}$ as “the moments.”

According to Liebert et al.,^16^ if there is a small change in the absorption coefficient of the sample at layer j, the moments experience a linear change given by

$$\Delta M_{n}=S_{n}^{j}\Delta \mu_{a}.$$ (2)

$S_{n}^{j}$ is the sensitivity of $M_{n}$ to changes in the absorption coefficient in layer j and is a function of the source–detector separation ρ associated with the measurement channel. These sensitivities can be estimated from Monte Carlo (MC) simulations (see Ref. 17).

Equation (2) can be used to model the total change in the moments of an fNIRS channel due to changes in absorption in different layers. It was proposed by Liebert et al.^16^ as part of a method to recover the evoked neural changes in the absorption coefficient for a two-layer tissue model (brain and scalp) from multidistance TD-fNIRS measurements with the following expression:

$$[\begin{array}{c} \Delta \mu_{a}^{\text{scalp}}\\ \Delta \mu_{a}^{\text{brain}} \end{array}]={\left(X^{T}Z^{-1}X\right)}^{-1}X^{T}Z^{-1}[\begin{array}{c} \Delta M_{0}\\ \Delta M_{1}\\ \Delta M_{2} \end{array}].$$ (3)

$\Delta \mu_{a}^{\text{scalp}}$ and $\Delta \mu_{a}^{\text{brain}}$ are the changes in the absorption coefficient of the superficial and the brain layers, respectively. $\Delta M_{n}$ are the column vectors containing the measured change in the moments for source–detector separations $\rho_{i}$ for i from 1 to $N_{s}$, where $N_{s}$ is the number of channels with different distances. X is a matrix containing the sensitivities for the different moments, distances, and layers and has a size of $3N_{s}\times 2$ (number of moments times the number of channels with different distances, by number of layers). Z is a covariance matrix of size $3N_{s}\times 3N_{s}$ containing the measurement covariances associated with each distance and moment. These covariances can be estimated from the DTOFs (either measured or estimated from MC simulations) assuming that the variance in the signal is dominated by shot noise, among other assumptions.^16^ All elements of Eq. (3) are wavelength-dependent.

## 3 Materials and Methods

### 3.1 Data Acquisition

We acquired 5-min long resting time series for six subjects using a Kernel flow TD-fNIRS system.^18^ Subjects were looking at a blank computer screen during acquisition. Measurements were obtained on consented subjects according to Boston University’s IRB 5965 and best practices.

**Fig. 1:**
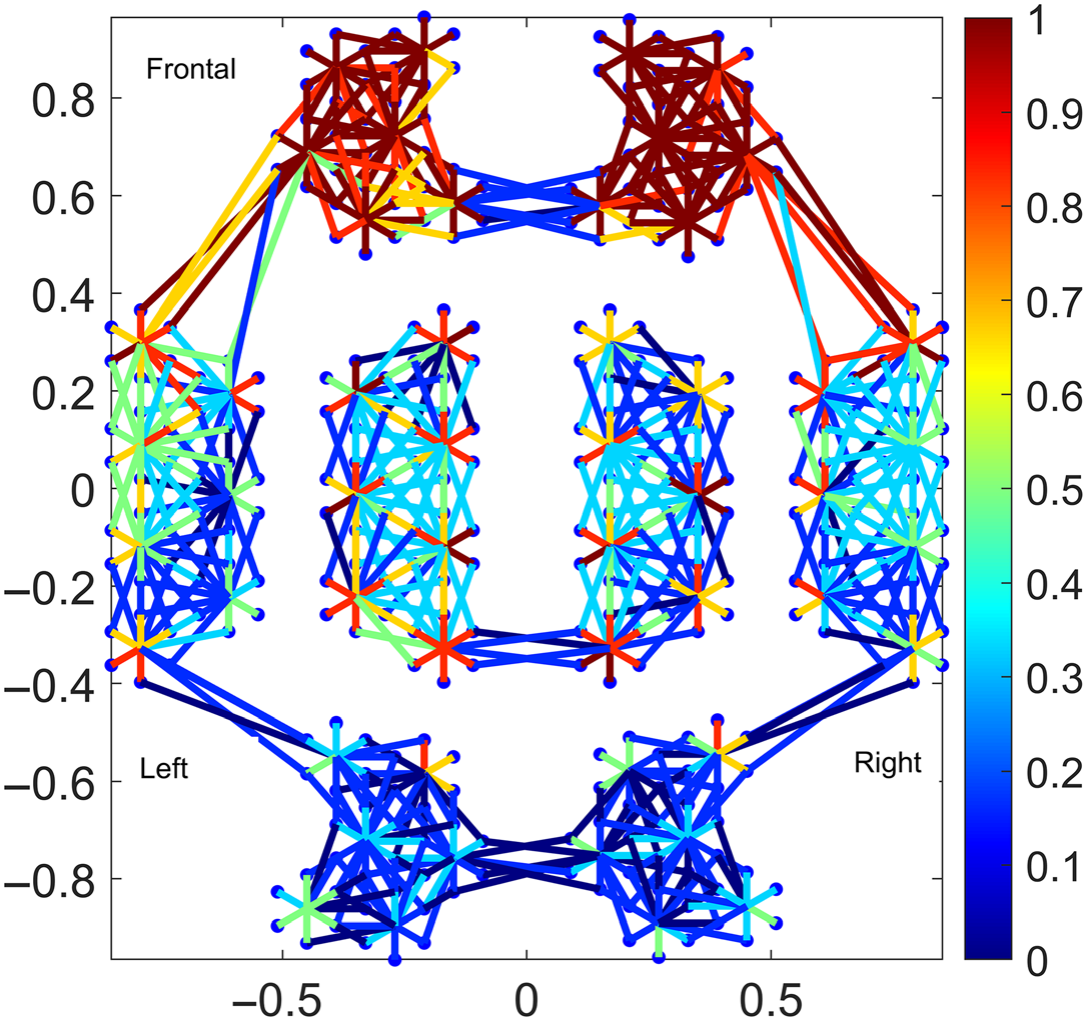
Diagram of the relative position of the channels on the head. This is a 2D projection as observed from above with the frontal part of the head on top. The channels are color coded according to how many of them were usable across subjects, with 1 representing all of them and 0 representing none. Channels longer than 34 mm were omitted for easier interpretation of the plot.

The Kernel flow^12^ is a TD-fNIRS system with almost complete full head coverage. Each module contains one source and six detectors and the whole montage consists of 52 sources (dual wavelength 690 and 850 nm) and 312 detectors, for a total of 2206 channels with source–detector separations ranging between 9.7 and 60 mm, acquired at 7 Hz. Of those, 312 are SS channels (<10  mm), 197 are medium separation (MS) channels (from 21 to 27 mm), 317 long separation (LS) channels (from 28 to 34 mm), and 1380 very long separation (VLS) channels (>34  mm). However, typically not all channels are usable due to experimental issues, such as hair pigmentation, hair density, and coupling to the head. On average, only 5.6% of the VLS channels of a subject were usable, as opposed to 73.9% of the SS channels. Figure 1 shows a 2D diagram of the position of the channels on the head (excluding the ones with a separation longer than 34 mm for clarity); the channels are color coded according to how many were usable on that region across subjects. Acquisition was performed with a customized computer using proprietary software provided by Kernel.

### 3.2 Data augmentation with Synthetic HRF

**Fig. 2:**
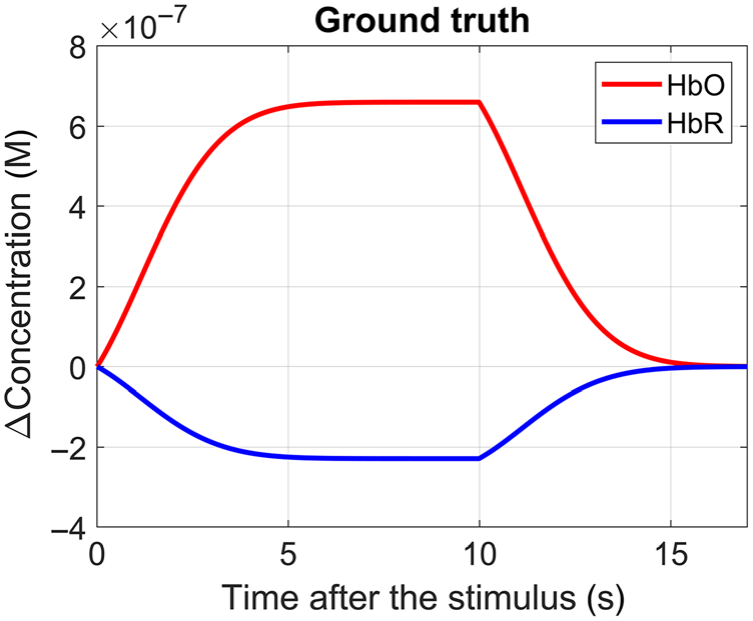
Synthetic HRF used to augment the time series (scale 1).

We augmented the moments of the channels from the resting time series with synthetic HRFs at random intervals. This was done by converting the HRF from Fig. 2 to changes in absorption coefficient using the Beer–Lambert law, and then using Eq. (2) for the appropriate source–detector distance to determine the change of moment to add to the resting moment time series (which was mean subtracted). The sensitivities $S_{n}^{\text{brain}}(\rho )$ were calculated from MC simulations assuming the superficial layer is 13-mm-thick (see Sec. 3.3). The added HRFs were spaced by a random interval with a mean of 21 s and standard deviation of 3 s. This way, each channel was augmented with 14 repetitions of the HRF. The HRF has an amplitude of about 0.6  μM for HbO and −0.2  μM for HbR. Only the channels with a source–detector separation <34  mm were augmented. Longer channels were excluded from analysis in this work as most of them did not present usable signals, which is consistent with the signal levels in other typical fNIRS devices.

**Fig. 3:**
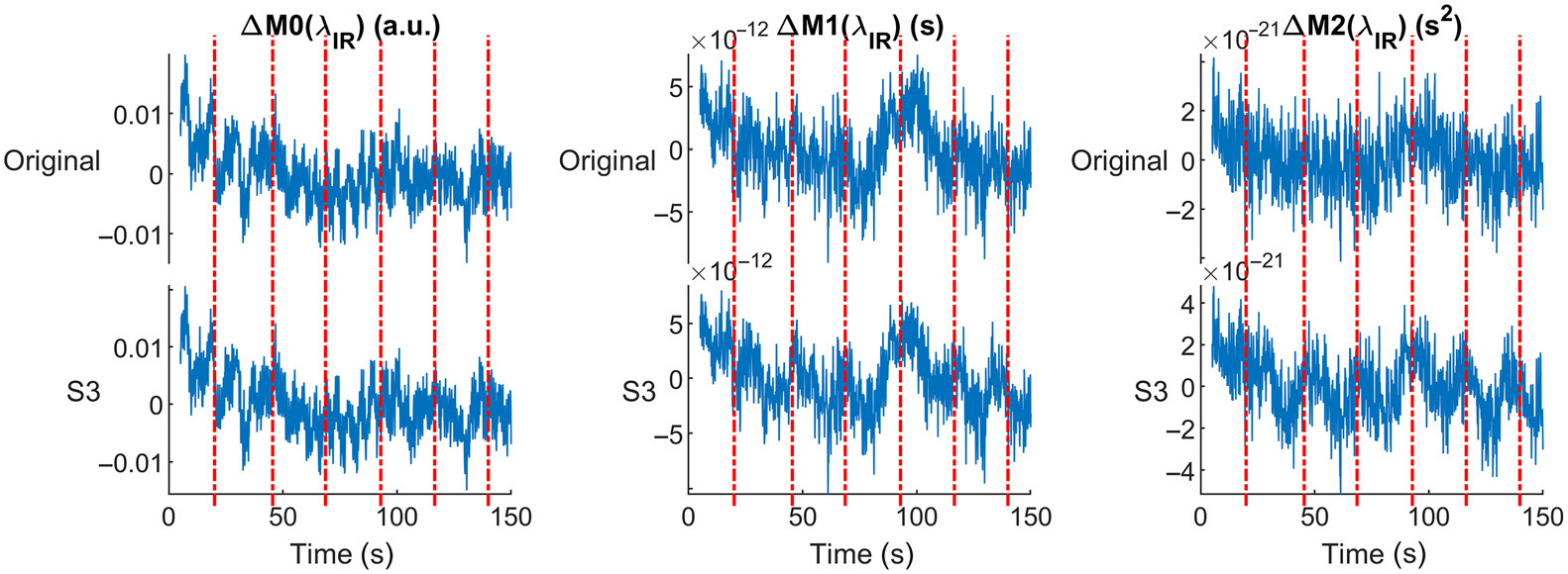
Comparison of original and augmented resting time series (for scale 3) at 850 nm. Notably, the added HRF is not obvious and the changes in the time series only become visually apparent for higher order moments, as the latter are more sensitive to changes in the absorption coefficient of the brain layer.

Additionally, we prepared additional augmented data with alternative amplitudes for the HRF. The alternative HRFs were obtained simply by scaling the HRF in Fig. 2 by 0.2, 0.5, and 3. We did this to investigate the effect of signal-to-noise ratio (SNR) on the HRF-recovering techniques. Most of the results on this work are for scale 1, unless noted otherwise. Figure 3 compares the original resting time series for one channel of one subject (at 850 nm) for all the three moments with the augmented time series of the same channel with scale 3.

### 3.3 Monte Carlo Simulations

We performed MC simulations of photons traveling from a source to a detector in multilayered media to calculate the sensitivity matrices for Eq. (2) as well as the shot-noise dominated covariance matrices for Eq. (3). These simulations were performed using Monte Carlo eXtreme.^19^^,^^20^ The simulations were used to calculate lookup tables for the sensitivities to changes in absorption coefficient for a range of scalp layer thicknesses, source–detector separations, and base absorption coefficients. These look-up tables can be quickly called during the augmentation of the resting data and for the recovery of the HRF.

The simulated volume was a 100×100×100  mm cube, with the source at the location (50, 50, 2) mm. The detector was placed at a distance ρ from the source, with simulations performed for all ρ ranging between 6 to 34 mm (in intervals of 2 mm). The simulation was performed with 20 layers. Layers 1 to 19 had a thickness of 1 mm. All layers had a scattering coefficient of $10\;{\mathrm{mm}}^{-1}$, anisotropy factor 0.9, and refraction index 1.4. We performed multiple simulations with different absorption coefficients from 0.009 to $0.03\;{\mathrm{mm}}^{-1}$ (in intervals of $0.001\;{\mathrm{mm}}^{-1}$). The simulated number of photons was $1\times 10^{8}$ with the max number of detected photons set as $1\times 10^{6}$.

Sensitivity values to each of the 20 layers for specific absorption coefficients and source–detector separations are calculated using linear interpolation from the closest values on this table. The sensitivity for the superficial layer is calculated by summing the sensitivities for the first $t_{\text{scalp}}$ layers, where $t_{\text{scalp}}$ is the thickness of the superficial layer in mm; similarly, the sensitivity for the brain layer is calculated by summing the sensitivities for the layers $t_{\text{scalp}}+1$ and beyond. The base absorption coefficient for the tissue was estimated assuming the tissue was 80% water and contained 30  μM of HbO and 20  μM of HbR (60% oxygen saturation).

The shot-noise-dominated covariances are also calculated from the MC simulations by first calculating the DTOFs for the base tissue, then calculating up to the fourth moment of the DTOFs. Then the moment for the specific source–detector separation and absorption coefficient are estimated through linear interpolation, which are then fed into Eqs. (12) to (17) from Liebert et al. ^16^ to calculate the variances. We called this covariance matrix calculated from theory $Z_{T}$.

### 3.4 Recovery of the HRF from TD Moments with Block Averaging

We recovered the HRF from the augmented resting data using the multidistance moment method described by Liebert et al.^16^ In this work, we will refer to this technique as TD moments. For each of the 312 detectors in the system, we selected 1 LS channel and 1 SS channel. The LS channel for each detector was chosen as the usable (good quality signal) channel with the source–detector separation closest to 30 mm (and <34  mm). Virtually, all detectors with a usable LS channel also had a usable SS channel (only 1% across all subjects did not). Detectors for which there were only usable SS channels were excluded from most of the analyses. The instrument returns “not-a-number” for channels with low-quality signal and thus those were considered unusable. All usable channels were considered “good quality” for our purposes.

We recovered the HRFs with the TD moment technique [Eq. (3)] using either just the LS channel or both the LS + SS channels (as the technique allows for multidistance). We will call these two TD LS and TD LS + SS methods, respectively. The TD moment technique uses Eq. (3) to estimate the absorption changes in the brain and the scalp layers from the measured change in the moments. After estimating the absorption changes in the brain, we performed block averages of these absorption changes according to the simulated stimuli timing. The block averages are then converted to changes in concentration of hemoglobin and compared to the ground truth.

It is necessary to estimate the covariance matrix Z to properly solve Eq. (3). We tried two approaches for estimating the covariance. The first was using the MC simulations with the equations by Liebert et al.^16^ to calculate the theoretical shot-noise dominated covariance $Z_{T}$ (see also Sec. 3.3). The second approach was to calculate the covariance directly from the measured time series for the moments. We first define the moment matrix:

$$Y_{M}=[\Delta M_{0}^{T},\Delta M_{1}^{T},\Delta M_{2}^{T}].$$ (4)

$\Delta M_{n}$ is a $N_{c}\times N_{t}$ vector ($N_{c}$ is the number of channels used for the multidistance analysis and $N_{t}$ is the number of time points sampled). $Y_{M}$ is of size $3N_{c}\times N_{t}$; the superscript T indicates a matrix transpose. In the case where two distances are used, i.e., both an LS and an SS channel, one of the two rows of $\Delta M_{n}$ is the measurements for the LS channel, and the other is the measurements for the SS channel. (Note: If performing a single-distance analysis, then $\Delta M_{n}$ is $1\times N_{t}$.) We can calculate an estimate of the covariance matrix as

$$Z_{E}=\mathrm{cov}(Y_{M},Y_{M}).$$ (5)

$Z_{E}$ is the covariance matrix estimated from the data and is of size $3N_{c}\times 3N_{c}$.

We used $Z_{T}$ and $Z_{E}$ to calculate the layer separation from Eq. (3) to compare the performance of each covariance scaling approach. Most of the results of TD techniques shown in this paper use the covariance estimated from the data $Z_{E}$, unless otherwise specified.

We used scaling when calculating Eq. (3) to improve the condition number of the matrices being inverted and thus to prevent numerical instability. For this, the estimators $X^{*}$ were calculated as

$$X^{*}={\left({\left(kX\right)}^{T}{\left(kZk^{T}\right)}^{-1}kX\right)}^{-1}{\left(kX\right)}^{T}{\left(kZk^{T}\right)}^{-1}k.$$ (6)

Which is algebraically equivalent to the standard weighted least squares expression given by $X^{*}={\left(X^{T}Z^{-1}X\right)}^{-1}X^{T}Z^{-1}$ and used in Eq. (3). Here k is a square diagonal matrix used to apply a weight to each moment so their units are in a similar scale. (The scales used were 1 for $M_{0}$, $1\times 10^{12}$ for $M_{1}$ and $1\times 10^{24}$ for $M_{2}$, but any set of values that put all the moments in similar order of magnitude should work.)

Additionally, we recovered the HRF with the TD moment technique using only one SS channel for each detector. We will call this TD SS. We did this to evaluate how TD SS compares to TD LS, as some past studies have shown that in TD-fNIRS the loss in brain sensitivity for the SS channels can be compensated by the much higher number of available photons in SS channels compared to LS channels.^21^

For comparison, we performed regular CW block averages of the augmented data by converting $\Delta M_{0}$ (which is equivalent to the CW optical density measurement) to changes in hemoglobin concentration using the modified Beer–Lambert law and then performing block averaging (after low-pass filtering at a 0.7-Hz cutoff frequency). We also applied the regular CW GLM technique to the augmented data for comparison of CW using LS and SS regression. The design matrix was built with Gaussian bases with spacing and standard deviation both equal to 1 s, plus the SS channel following the method described in Gagnon (2011, static estimator section ^22^). No polynomial drift terms were used, and no delays were applied to the SS channel.

We calculated the RMSE and the Pearson correlation between the recovered HRF and the ground truth for each detector to compare the performance of the different recovery techniques. The recovered HRF were baseline adjusted by subtracting the mean of the HRF from −2 to 0 s. The Pearson correlation was Fisher-transformed to convert it to a normal distribution. The Fisher transform is given by the following expression:

$$z=\frac{1}{2}\;\ln(\frac{1+r}{1-r}),$$ (7)

where r is the Pearson correlation coefficient and z is the Fisher-transformed Pearson correlation. For the rest of this work, all presented correlations will be Fisher transformed. RMSEs and correlations were compared between processing groups using paired Student t-tests (α=0.05).

### 3.5 GLM for TD Moments

In addition to processing the augmented data with the TD moment technique, we also propose, implement, and demonstrate a GLM to analyze TD fNIRS moments that also simultaneously models the confounding physiological signals in the scalp. For the rest of this work, we will call this technique “TD GLM.” TD GLM is based on the supposition that the change in the n’th moment observed in a given channel is the linear superposition of a change in the moment caused by the hemodynamic changes in the brain and a change in moment caused by the physiology and other artifacts, and that the latter is predominantly observed in the scalp, i.e.,

$$\Delta M_{n}^{\mathrm{LS}}(\lambda ,t)=\beta_{\mathrm{SS}}(\lambda )\Delta M_{n}^{\text{scalp}}(\lambda ,t)+\Delta M_{n}^{\text{brain}}(\lambda ,t).$$ (8)

Here $\Delta M_{n}^{\mathrm{LS}}(\lambda ,t)$ is the measured change in the n’th moment for an LS channel as a function of time observed for wavelength λ. $\Delta M_{n}^{\text{scalp}}(\lambda ,t)$ is the contribution of the scalp/superficial layer to the change in the moment, whereas $\Delta M_{n}^{\text{brain}}(\lambda ,t)$ is the contribution of the brain layer. $\beta_{\mathrm{SS}}(\lambda )$ is a coupling constant. From Eq. (2), the change in moment from the scalp layer of the first term of Eq. (8) can be expressed as

$$\Delta M_{n}^{\text{scalp}}(\lambda ,t)=S_{n}^{\text{scalp}}(\lambda ,\rho_{LS})\Delta \mu^{\text{scalp}}(\lambda ,t).$$ (9)

Here $S_{n}^{\text{scalp}}(\lambda ,\rho_{\mathrm{LS}})$ is the sensitivity to changes in absorption coefficient of the scalp for the LS channel, and $\Delta \mu^{\text{scalp}}(\lambda ,t)$ is the change in absorption coefficient on the scalp layer. Now, assume that we have an SS measurement of the change in the zeroth moment $\Delta M_{0}^{SS}(\lambda ,t)$. As the zeroth moment has low sensitivity to the brain layer compared to the scalp layer, we can use Eq. (2) to make the approximation:

$$\Delta M_{n}^{\text{scalp}}(\lambda ,t)\approx S_{n}^{\text{scalp}}(\lambda ,\rho_{\mathrm{LS}})\frac{\Delta M_{0}^{\mathrm{SS}}(\lambda ,t)}{S_{0}^{\text{scalp}}(\lambda ,\rho_{\mathrm{SS}})}.$$ (10)

Here $S_{0}^{\text{scalp}}(\lambda ,\rho_{\mathrm{SS}})$ is the sensitivity to changes in absorption coefficient on the scalp layer for the SS channel for the zeroth moment. Similarly, the second term of Eq. (8) can be expressed as

$$\Delta M_{n}^{\text{brain}}(\lambda ,t)=S_{n}^{\text{brain}}(\lambda ,\rho_{\mathrm{LS}})(\epsilon_{\mathrm{HbO}}(\lambda )\Delta C_{\mathrm{HbO}}^{\text{brain}}(t)+\epsilon_{\mathrm{HbR}}(\lambda )\Delta C_{\mathrm{HbR}}^{\text{brain}}(t)),$$ (11)

where the change in absorption coefficient of the brain layer was expressed as changes in the concentration of hemoglobin with the Beer–Lambert law. $S_{n}^{\text{brain}}(\lambda ,\rho_{\mathrm{LS}})$ is the sensitivity to changes in absorption coefficient of the brain layer of the LS channel for the n’th moment. $\epsilon_{\mathrm{HbX}}(\lambda )$ are the extinction coefficients corresponding to the hemoglobin species, whereas $\Delta C_{\mathrm{HbX}}^{\text{brain}}(t)$ is their change in concentration in the brain layer. Now, we will express the changes in hemoglobin concentration as a linear combination of $N_{\beta }$ temporal basis functions $g_{k}(t)$:

$$\Delta C_{\mathrm{HbX}}^{\text{brain}}(t)=\overset{N_{\beta }}{\underset{k=1}{\sum }}\beta_{k}^{\mathrm{HbX}}g_{k}(t).$$ (12)

Combining Eqs. (8) and (11) with Eq. (12):

$$\Delta M_{n}^{\mathrm{LS}}(\lambda ,t)=\beta_{\mathrm{SS}}(\lambda )\Delta M_{n}^{\text{scalp}}(\lambda ,t)+S_{n}^{\text{brain}}(\lambda ,\rho_{\mathrm{LS}})(\epsilon_{\mathrm{HbO}}(\lambda )\overset{N_{\beta }}{\underset{k=1}{\sum }}\beta_{k}^{\mathrm{HbO}}g_{k}(t)+\epsilon_{\mathrm{HbR}}(\lambda )\overset{N_{\beta }}{\underset{k=1}{\sum }}\beta_{k}^{\mathrm{HbR}}g_{k}(t)).$$ (13)

Equation (13) is a linear model for the LS channel as a sum of temporal basis functions and the change in the moment caused by the scalp modeled by Eq. (10). If we have a series of measurements in time of the change in moment, we can express this in matrix form as

$$\Delta M_{n}^{\mathrm{LS}}=U_{n}\underset{\_}{\beta }.$$ (14)

Here $\Delta M_{n}^{\mathrm{LS}}$ is a column vector of the measurements of the change in the n’th moment observed for the LS channel and has a size of $2N_{t}\times 1$ ($N_{t}$ being the number of time samples). $\underline{\beta }$ is a column vector containing the regression coefficients and has a length of $2N_{\beta }+2$. $U_{n}$ is the design matrix for moment n [size is $2N_{t}\times (2N_{\beta }+2)$]. Explicitly, $\underset{\_}{\beta }$ is

$$\underset{\_}{\beta }=[\begin{array}{c} \beta_{\mathrm{SS}}(\lambda_{1})\\ \beta_{\mathrm{SS}}(\lambda_{2})\\ \beta_{1}^{\mathrm{HbO}}\\ \beta_{1}^{\mathrm{HbR}}\\ \beta_{2}^{\mathrm{HbO}}\\ \beta_{2}^{\mathrm{HbR}}\\ \ldots \end{array}].$$ (15)

The design matrix for moment n is

$$U_{n}=[\begin{array}{cccccc} \Delta M_{n}^{\text{scalp}}(\lambda_{1},\underset{\_}{t}) & 0 & G_{n,1}^{\mathrm{HbO}}(\underset{\_}{t},\lambda_{1}) & G_{n,1}^{\mathrm{HbR}}(\underset{\_}{t},\lambda_{1}) & G_{n,2}^{\mathrm{HbO}}(\underset{\_}{t},\lambda_{1}) & \ldots \\ 0 & \Delta M_{n}^{\text{scalp}}(\lambda_{2},\underset{\_}{t}) & G_{n,1}^{\mathrm{HbO}}(\underset{\_}{t},\lambda_{2}) & G_{n,1}^{\mathrm{HbR}}(\underset{\_}{t},\lambda_{2}) & G_{n,2}^{\mathrm{HbO}}(\underset{\_}{t},\lambda_{2}) & \ldots \end{array}].$$ (16)

And $\Delta M_{n}^{\mathrm{LS}}(\underline{t})$ is

$$\Delta M_{n}^{\mathrm{LS}}(\underline{t})=[\begin{array}{c} \Delta M_{n}^{\mathrm{LS}}(\lambda_{1},\underline{t})\\ \Delta M_{n}^{\mathrm{LS}}(\lambda_{2},\underline{t}) \end{array}].$$ (17)

With $\Delta M_{n}^{\mathrm{LS}}(\lambda ,\underline{t})$ being the measured changes of moment n in the LS channel for wavelength λ. We defined the scaled basis functions $G_{n,k}^{\mathrm{HbX}}(\underline{t},\lambda )$ as

$$G_{n,k}^{\mathrm{HbX}}(\underline{t},\lambda )=S_{n}^{\text{brain}}(\lambda ,\rho_{\mathrm{LS}})\epsilon_{\mathrm{HbX}}(\lambda )g_{k}(\underline{t}).$$ (18)

Now, if we measured the changes for moments zero to two, we could combine all measurements in a matrix from Eq. (14):

$$[\begin{array}{c} \Delta M_{0}^{\mathrm{LS}}(\underset{\_}{t})\\ \Delta M_{1}^{\mathrm{LS}}(\underset{\_}{t})\\ \Delta M_{2}^{\mathrm{LS}}(\underset{\_}{t}) \end{array}]=[\begin{array}{c} U_{0}\\ U_{1}\\ U_{2} \end{array}]\underset{\_}{\beta }.$$ (19)

Next, we define

$$Y=[\begin{array}{c} \Delta M_{0}^{\mathrm{LS}}(\underset{\_}{t})\\ \Delta M_{1}^{\mathrm{LS}}(\underset{\_}{t})\\ \Delta M_{2}^{\mathrm{LS}}(\underset{\_}{t}) \end{array}]$$ (20)

and

$$U=[\begin{array}{c} U_{0}\\ U_{1}\\ U_{2} \end{array}].$$ (21)

Then we can solve for the regression coefficients $\underset{\_}{\beta }$ in Eq. (19) as

β^_=(UTC−1U)−1UTC−1Y, (22)

where C is the covariance matrix of the measurements. C is of size $6N_{t}\times 6N_{t}$ and can be calculated from theory if shot-noise dominated. Alternatively, if we assume no serial correlation in the channels (white noise), we can estimate it from the measured data as

$$C_{E}=\mathrm{cov}(Y_{M},Y_{M})⊗I(N_{t}),$$ (23)

where ⊗ represents the Kronecker product and $I(N_{t})$ is the identity matrix of order $N_{t}$. $\mathrm{cov}(Y_{M},Y_{M})$ is the covariance matrix of matrix $Y_{M}$. $Y_{M}$ is defined as

$$Y_{M}=[\Delta M_{0}^{\mathrm{LS}}(\lambda_{1},\underset{\_}{t}),\Delta M_{0}^{\mathrm{LS}}(\lambda_{2},\underset{\_}{t}),\Delta M_{1}^{\mathrm{LS}}(\lambda_{1},\underset{\_}{t}),\Delta M_{1}^{\mathrm{LS}}(\lambda_{2},\underset{\_}{t}),\Delta M_{2}^{\mathrm{LS}}(\lambda_{1},\underset{\_}{t}),\Delta M_{2}^{\mathrm{LS}}(\lambda_{2},\underset{\_}{t})].$$ (24)

$Y_{M}$ has size of $N_{t}\times 6$ [and analogous to $Y_{M}$ in Eq. (4)]. For the rest of this work, we will call the covariance matrices C used for Eq. (22) as Z, in analogy to the covariance matrices used in Sec. 3.4. In fact, the C matrices are the Z matrices after a Kronecker product with the appropriate identity matrix.

We used Eq. (22) to recover the HRF from the augmented time series. We used sparse matrix techniques to increase computational efficiency, as the matrices to be inverted can become very large depending on the number of time samples acquired. Most of the results shown in this paper were calculated with the covariance estimated from the data [Eq. (23)], unless otherwise specified. All data processing was performed with custom MATLAB scripts.

## 4 Results

**Fig. 4:**
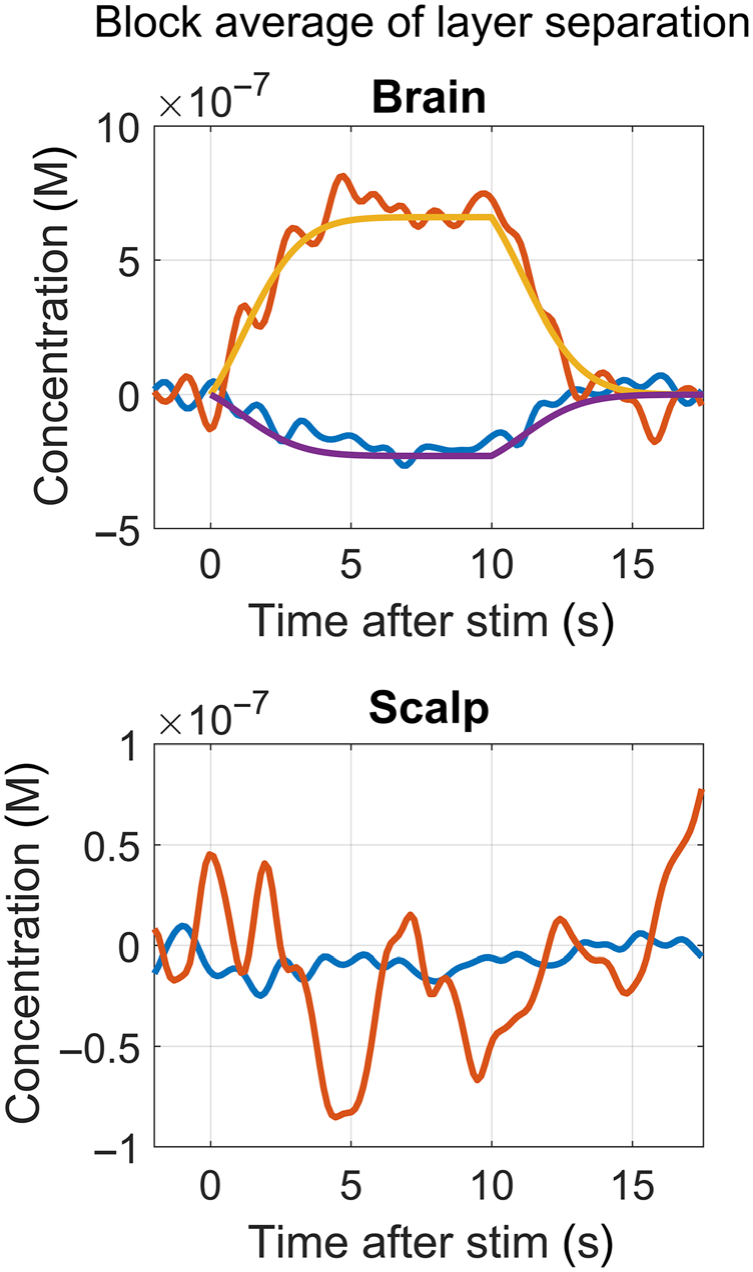
Example of HbO (red) and HbR (blue) block averages for one channel, in which we applied multidistance TD moment analysis to separate brain from scalp. The top figure also shows the ground truth (orange for HbO and purple for HbR).

Figure 4 shows an example of the block average HbO (red) and HbR (blue) obtained from the brain layer (top) and the superficial layer (bottom) separated with the TD (LS + SS) moment analysis. This is for one channel of one subject located on the prefrontal cortex. The HbO curves are in red and the HbR in blue. The top panel also shows the ground truth of the HRF in orange and purple (HbO and HbR, respectively).

**Fig. 5:**
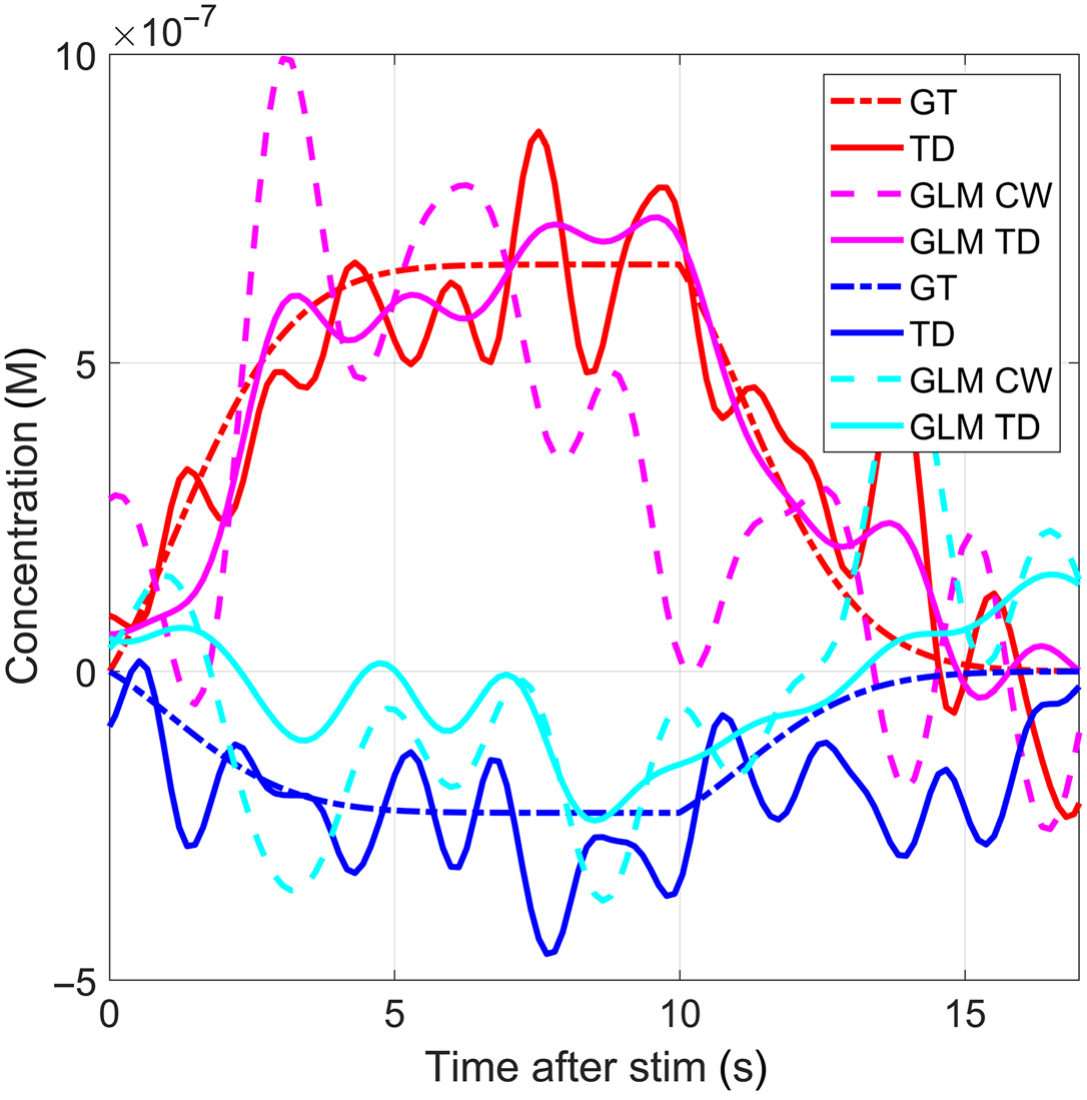
Example of recovered HRFs and their ground truths obtained with different techniques, mean across all subjects for channel 14.

Figure 5 shows an example of the HRF recovered with several of the techniques used in this paper; here GT is the ground truth, TD is the TD (LS + SS) moment technique (described in Sec. 3.4), GLM CW is the traditional GLM calculated from CW ($M_{0}$) data only, and GLM TD is the time-domain moment GLM proposed on Sec. 3.5. The HRFs displayed for each technique are an average across subjects for one of the channels (channel 14).

**Fig. 6:**
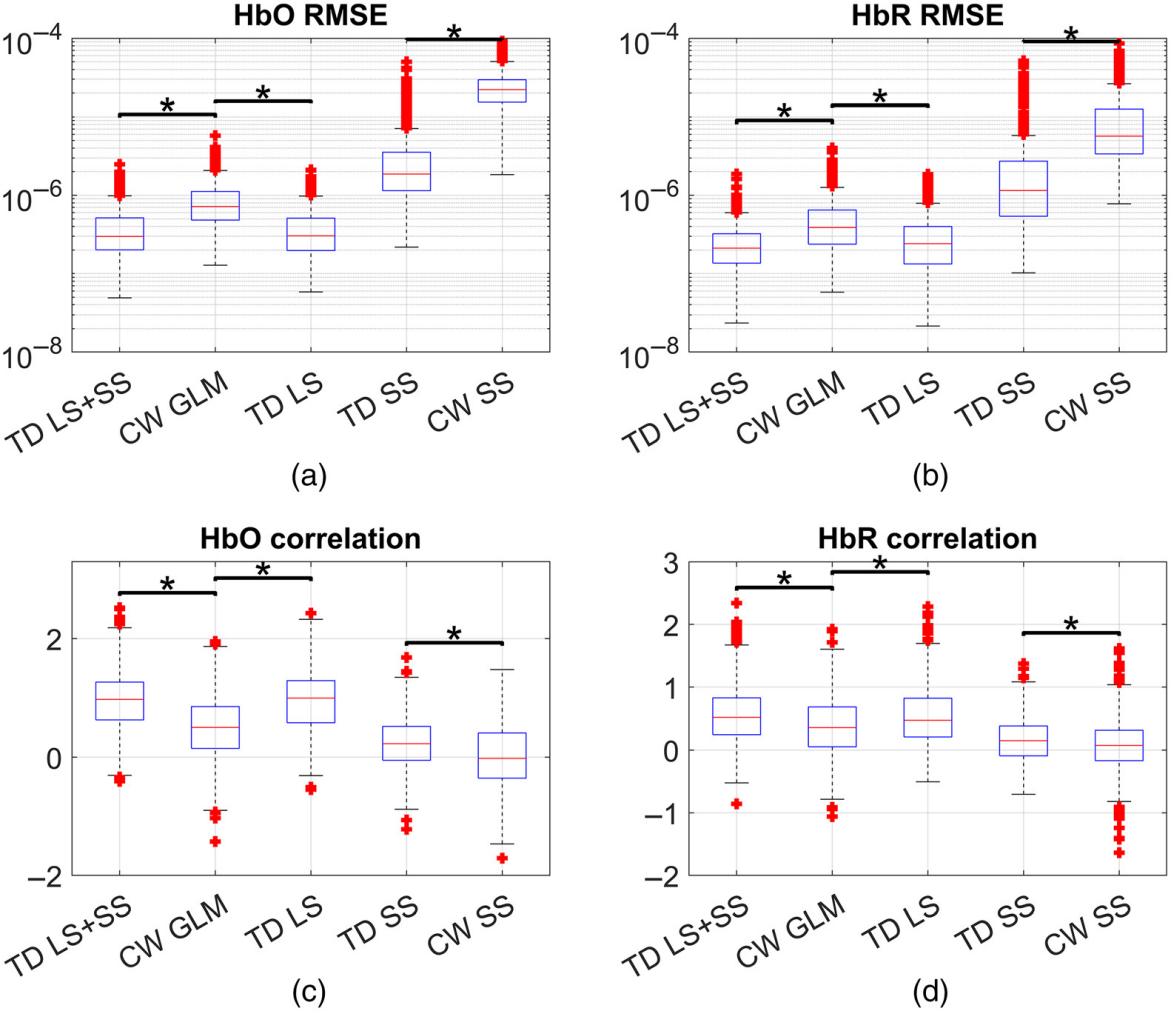
(a), (b) RMSE and (c), (d) Fisher-transformed correlation for (a), (c) HbO and (b), (d) HbR across all subjects and channels for TD-moment analysis versus block averaging CW and GLM (LS + SS). The results are segregated by source–detector separation range, where LS: long separation, MS: medium separation, and SS: short separation.

Figure 6 shows box plots comparing the RMSE and Fisher-transformed correlations obtained from the recovered HRF for all subjects and channels, both for HbO and HbR. TD LS + SS is the multidistance TD moment analysis, CW GLM is the traditional CW GLM with SS regression. TD LS is the moment analysis technique with only one distance (LS). We also included TD SS (moment analysis technique with single distance, SS) and CW SS (block average for CW data for the SS channel). The TD techniques outperform their CW equivalents for all chromophores and metrics (α=0.05). We failed to find a statistically significant difference for TD LS + SS and TD LS (α=0.05) for HbR correlation and HbO RMSE, indicating that the addition of the SS information in general did not significantly improve the performance of the LS in time domain.

**Fig. 7:**
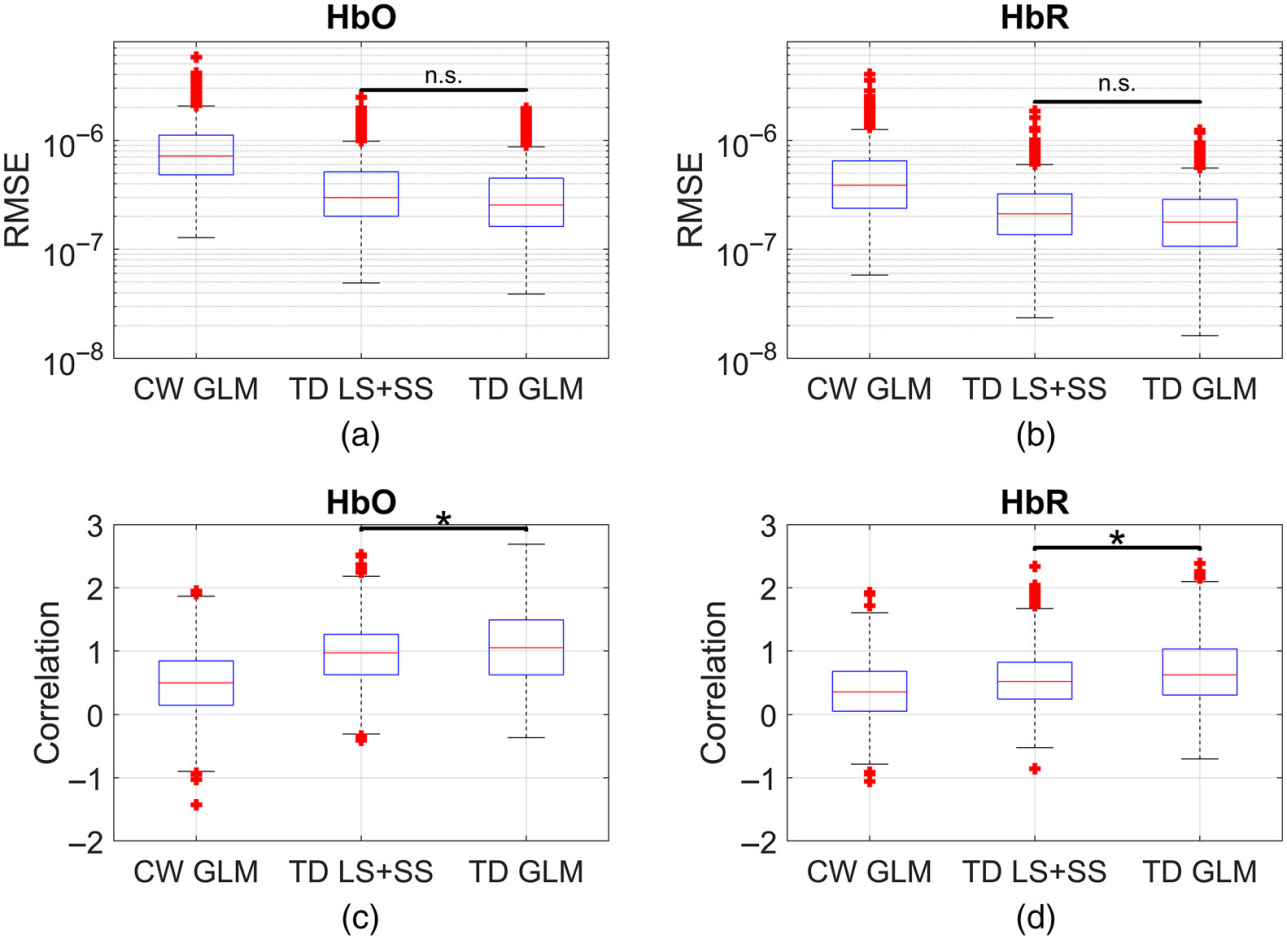
Comparison of the performance of GLM (CW and TD) with simple two distance moment analysis for both (a), (c) HbO and (b), (d) HbR across all subjects and channels.

Figure 7 compares the performance of the TD GLM to the TD moment analysis and includes CW GLM for comparison. The results shown are across all subjects and channels. The TD GLM performs statistically better than TD moment analysis in correlation but not in RMSE. On average, TD moment HRF recovery improves Fisher-transformed correlations by 98% and 48% for HbO and HbR, respectively, over CW GLM. The improvement on the Fisher-transformed correlation for TD GLM over TD moment is 12% (HbO) and 27% (HbR). RMSE decreases 56% and 52% (HbO and HbR) for TD moment compared to CW GLM.

**Fig. 8:**
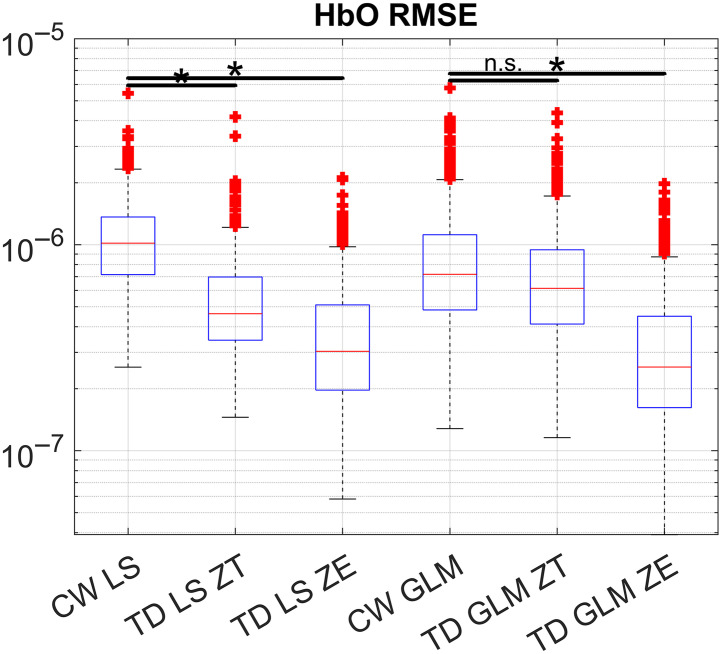
Covariance scaling (ZT versus ZE, theoretical and estimated from measurements, respectively) affects the HbO RMSE, for block averaging techniques (TD LS) and for GLM techniques (TD GLM). The equivalent CW technique results were included for comparison.

Figure 8 is used to exemplify the effects of the choice for the covariance scaling on the HbO RMSE across all subjects and channels. The three blocks on the left side of this figure are block averaging techniques, with CW LS being CW block averaging of the LS channels (included for reference), TD LS ZT is the TD moment analysis for the LS channels using the theoretical shot-noise dominated covariance ($Z_{T}$), and TD LS ZE is the TD moment analysis using the covariance estimated from the data ($Z_{E}$). Similarly, the three boxes on the right are for the GLM techniques, starting with the simple CW GLM, then ZT TD GLM scaled with the theoretical shot-noise dominated covariance, and TD GLM ZE is the TD GLM scaled with the covariance estimated from the noise.

**Fig. 9:**
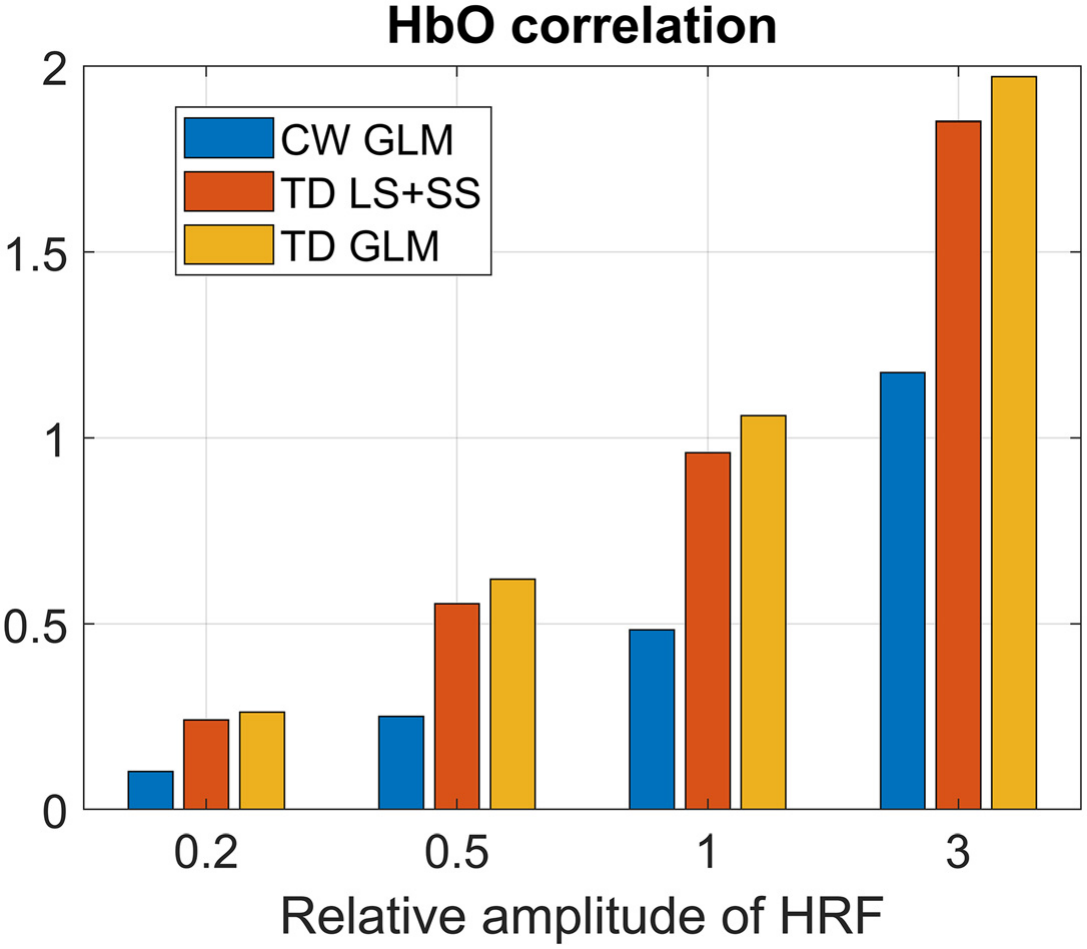
Comparison of the mean Fisher-transformed correlation across all subjects and detectors for HbO at different HRF scales. Similar performance rankings are observed for the other chromophores and for the RMSE.

Figure 9 shows an analysis of how the amplitude of the HRF affects the HbO correlation to ground truth for the TD techniques, with the CW GLM included for comparison. It shows how the relative amplitude of the added HRF affects the performance of different techniques as measured with the Fisher transformed correlation.

## 5 Discussion

### 5.1 CW Techniques versus Moment Techniques

Although alternative fNIRS acquisition techniques, such as TD fNIRS and FD (frequency domain) fNIRS,^23^ offer many theoretical advantages over the more traditionally used CW fNIRS (including enhanced sensitivity to deeper tissue^24^^,^^25^), several practical aspects have hindered their adoption. In particular, TD and FD fNIRS require much more complicated and precise (and thus, potentially more expensive) instrumentation, as well as more sophisticated processing techniques to reach their full potential. For these reasons, there has only been a very limited number of commercial devices released for FD and TD compared to those for CW fNIRS. To our knowledge, only one company (ISS, Illinois) offers a commercial FD system and another one (Hamamatsu, Japan) a commercial TD system, both with a very limited channel number. Meanwhile, CW fNIRS devices are offered by around a dozen companies, and they have been increasing their channel number offerings to a point that full head coverage and high density are starting to become routine. This has produced a relative lack of researcher experience for TD and FD, making it difficult to determine if the advantages over CW fNIRS can be fully realized. For example, the simplifying assumptions about tissue geometry and composition might compromise FD fNIRS ability to accurately measure absolute changes in the concentration of the chromophores.^23^^,^^26^ Similarly, the effect of the IRF on TD fNIRS compromises the correct discrimination of the photons coming from the different tissue layers. In that sense, moment analysis has been proposed as an alternative to TD fNIRS analysis that is relatively immune to the effects of the IRF. The results of this work support that moment analysis of TD fNIRS time series outperforms equivalent CW fNIRS techniques in recovering the functional hemodynamic neural response due to increased sensitivity to deeper tissue layers. However, there are some caveats to this improvement in performance.

The first caveat is that TD moment analysis depends on calculations derived from MC simulations. MC simulations are generally too slow to be performed at the time of the analysis. However, as we did in this work, it is possible to perform the MC simulations ahead of time for a range of typical parameters, then to precalculate the sensitivities and store them as look-up tables. The required quantities can be quickly accessed later during the estimation of the HRF. Nevertheless, the accuracy of the TD moment analysis will depend on the estimations of the sensitivities, and thus it might be worth investigating the error introduced by Monte Carlo models being inaccurate to the real tissue being measured.

TD techniques using moments are much more sensitive to numerical instability than equivalent CW techniques. This is mainly because each moment is in units at a much different scale ($M_{0}$ is on the order of magnitude of $1\times 10^{0}$, $M_{1}$ is $1\times 10^{-12}\;s$, and $M_{2}$ is $1\times 10^{-24}\;s^{2}$). Covariance scaling can stabilize this, but the inversion of Z in Eq. (3) might require additional scaling to avoid inaccurate results. Furthermore, covariance scaling with Z is crucial for obtaining good results when dealing with TD moments, as it converts the different moments to comparable scales while assigning them a relative importance/weight. For example, Fig. 8 shows that an improper selection of the covariance scaling can make the TD GLM not any better than the CW GLM. Although covariance scaling can also be used in CW techniques, the effect is much smaller as the optical density for the different wavelengths tends to be of a similar order of magnitude and have similar measurement variances relative to that of the different moments.

Figure 6 shows that TD moment SS has a much better performance than CW SS due to the higher sensitivity to the brain layer of the higher order moments. Furthermore, in terms of RMSE, TD SS rivals the performance of the CW GLM (and seems to be slightly better in terms of correlation). This suggests that it could be feasible to build relatively inexpensive TD fNIRS systems that only measure SS channels but still outperform CW fNIRS. In fact, as moments are less sensitive to the IRF than using time gates in TD,^14^ engineers designing affordable TD fNIRS system may not need to optimize the IRF and can thus keep costs low.

TD NIRS systems have traditionally been much more expensive, heavier, and bulkier than equivalent CW systems. As a consequence, literature on its applications has been mostly limited to tissue oxygenation,^27^^,^^28^ tissue imaging (including mammography^29^^,^^30^), and some proofs of concept of neural functional responses to tasks,^31^^,^^32^ though multiple other applications have been proposed across the years.^33^^,^^34^ However, the release of the Kernel flow prototype has demonstrated that current technology allows the implementation of TD fNIRS systems with a relatively small footprint and at lower cost than traditional TD systems. Even then, the Kernel flow consumes a considerable amount of power and the helmet alone weights more than four pounds, limiting its mobility, especially compared to the recent CW systems, which are trending toward wearability,^35^^,^^36^ affordability, and open source.^37^ Despite this, it is expected that TD fNIRS technology will continue progressing in usability and cost, and there are efforts toward developing more portable implementations of the technology,^38^ which might result in innovative clinical and practical applications (such as identification of intoxication biomarkers^39^).

### 5.2 Estimation of the Covariance Weights

As previously mentioned, proper estimation of the covariance weights is crucial when analyzing TD moments. Our first approach was to use the theoretical covariance described in the literature, but it did not yield the best results for several reasons. First, those calculations assume that we know the number of photons received by the detector, which we do not necessarily know without characterization of the hardware. Second, the theoretically calculated covariance was overestimating the covariance of the higher order moments compared to the lower order moments, giving the former comparatively smaller weight in the estimation. This is because the theory assumed that the variance is shot noise dominated in order to make simplifying assumptions that would allow the derivation of simple expressions to calculate the variance from moments of the HRF. In practice, real data have sources of variance other than shot noise. Importantly, variance caused by physiology affects the upper layer more than the deeper layers and consequently and affects the higher order moments less as they are less sensitive to the upper layer relative to the deeper layers.

We assumed no serial correlations when calculating the TD GLM (white noise assumption). This is not realistic as periodic physiology and other artifacts produce temporal correlations.^9^ However, this becomes less of a problem if the GLM appropriately models these sources of temporal correlation.

We obtained much better results using the covariances calculated straight from the measured time series (as seen in Fig. 8). This is because the statistical distribution of the measurement noise does not satisfy the assumption of it being shot noise dominated. Even then, our estimation of the covariance as the sample covariance of the data $Z_{E}$ has limitations, as the time series contain nonstationary sources of noise (e.g., motion artifacts^9^). Thus better estimations of the covariance (e.g., by recursive methods^40^) could provide additional improvements for time-domain techniques.

### 5.3 TD GLM versus Multidistance TD Moment Analysis

TD GLM outperforms CW GLM and multidistance moment analysis for LS + SS. However, multidistance TD moment analysis with three or more distances has the potential to outperform the TD GLM (see Fig. S1 in the Supplementary Material). As multidistance analysis is simpler and less computationally expensive than TD GLM, there might be cases where it is preferable to use simple multidistance TD moment analysis. However, it is not always possible to have multidistance measurements, due to either hardware or experimental limitations (i.e., not all channels having a good SNR). In fact, traditional fNIRS systems used only one fixed source–detector separation (30 mm), and customizable distance and SS channels are only becoming more popular in recent years.

The multidistance TD moment analysis assumes that all the channels used in the analysis are observing the same change in absorption coefficient in the brain layer, which is not necessarily realistic if the changes are spatially slanted toward one of the channels and away from the others (our analysis assumed all channels associated with a detector were observing the same region of the brain layer). The GLM does not require this assumption, and in fact, works better if the SS channel contains no contribution from the HRF. Incidentally, this is the reason our TD GLM model only uses $M_{0}$ for SS, as higher order moments have higher contributions of the brain layer and thus using them to model the superficial layer yields worse results.

Furthermore, there is a selection bias for the multidistance analysis since there are a limited number of detectors that had more than two different distances available, and those had better SNR than the channels in other detectors. Thus the mean results for TD moment with three or more distances are for a limited number of detectors with higher average quality channels, whereas the statistics for the other techniques (LS and LS + SS) are averaging good and bad quality channels.

We found a statistically significant improvement of the TD GLM over TD LS + SS for the correlation but not for the RMSE. However, we could argue that correlation is a better metric than RMSE as it is independent of scale. RMSE also has the disadvantage of being highly dependent on the method used to remove the baseline from the estimated HRF.

Another argument for the TD GLM over the (multidistance) TD moment analysis is that the performance of the TD GLM could potentially be improved further over what we obtained by multimodal regression incorporating relevant auxiliary signals (e.g., accelerometers to remove motion artifacts^41^). A different choice of temporal bases might also benefit some cases, for example, using the canonical gamma function in cases that merit it, which has the effect of reducing the degrees of freedom of the regression problem. Additionally, autoregressive techniques could be used to solve the TD GLM.^42^

Unlike the regular CW GLM formulation, our TD GLM integrates the information for the two wavelengths in one model, avoiding the necessity to perform a separate calculation for oxyhemoglobin and for deoxyhemoglobin (and thus the model has all the available information and not just half of it). Furthermore, this model performs the conversion from moments to concentration (which incorporates the available knowledge about the differential pathlength factor or sensitivity), requiring one less preprocessing step.

Finally, for cases where there are multiple overlapping channels with multiple distances, it might be better to develop an image reconstruction technique that incorporates moment analysis instead of using multidistance techniques, as image reconstruction techniques model the spatial distribution of the hemodynamic perturbations better.^43^^,^^44^

### 5.4 Effect of HRF Amplitude

The analysis with multiple HRF amplitudes showed that even at different scales, TD techniques outperform CW techniques. The pattern of TD moment LS + SS outperforming the CW GLM and the TD GLM outperforming TD moment LS + SS holds for all the used scales for HbO and HbR correlations (the latter not shown). Similar patterns are observed for the RMSE (HbO RMSE is shown in Fig. S2 in the Supplementary Material). The CW GLM seems to perform relatively worse for lower amplitudes of the HRF, as the correlation difference between CW GLM and TD GLM grew smaller for larger amplitudes.

## 6 Conclusions

In this work, we compared TD-fNIRS moment techniques with equivalent CW fNIRS techniques in terms of their performance in the estimation of the HRF from augmented resting time series. We found that, as long as adequate covariance scaling is used and measures are taken to prevent numerical instabilities, TD-moment techniques outperform their CW counterparts in RMSE and correlation to ground truth. Furthermore, we propose a GLM for TD-moments that further improve the performance over previously reported nonmodel-based LS + SS TD-moments fitting by allowing incorporation of knowledge of other auxiliary measurements, such as independent measures of confounding physiological signals from the scalp.
