# Supplementary material for: How much do time-domain functional near-infrared spectroscopy (fNIRS) moments improve estimation of brain activity over traditional fNIRS?
Source: Neurophotonics. 2022 Oct 22;10(1):013504. doi: 10.1117/1.NPh.10.1.013504 (PMC9587749; doi:10.1117/1.NPh.10.1.013504)
Supplement: Supplementary file 1 [file NPh_010_013504_SD001.pdf]

**Supplemental figure and captions**

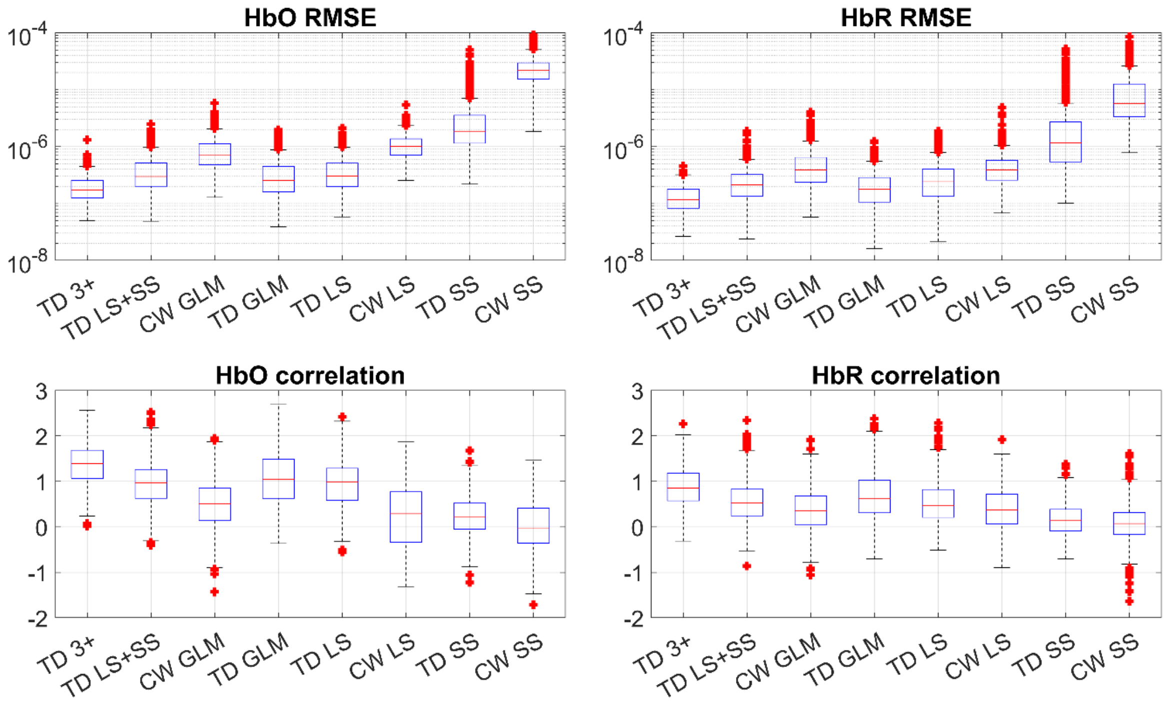


Figure S1: RMSE and Fisher transformed correlation for HbO and HbR for all subjects and channels for various techniques. TD 3+ is multi-distance TD moment analysis with 3 (or 4, when available) source detector separations. TD LS + SS is multi-distance TD moment with one LS and one SS channel. CW GLM and TD GLM are the respective GLMs with LS and short separation regression. TD LS is TD single distance moment analysis for LS channels. CW LS is block averaging of CW for LS channels. Similarly, TD SS and CW SS are for single distance SS channels

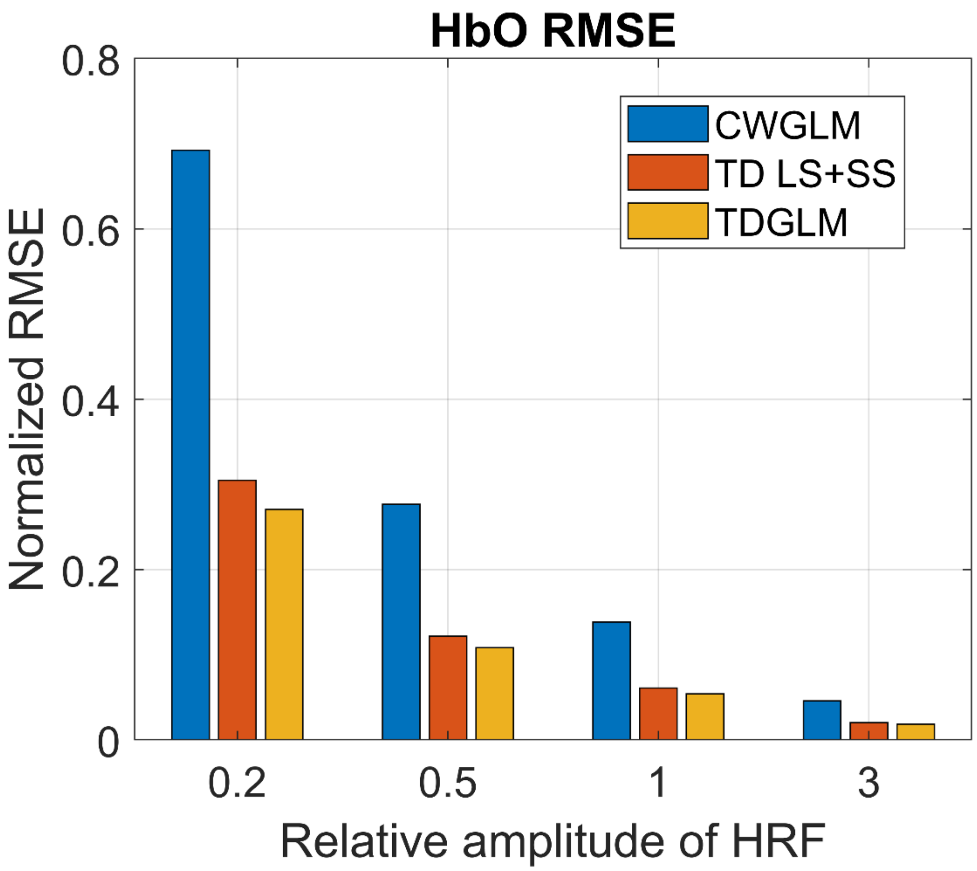


Figure S2: Comparison of the mean HbO RMSE across all subjects and channels for different amplitudes of the HRF. The RMSE has been normalized to the absolute value of the area under the curve of the ground truth for easier comparison
